# Human Endogenous Retrovirus Group E and Its Involvement in Diseases

**DOI:** 10.3390/v7031238

**Published:** 2015-03-16

**Authors:** Christelle Le Dantec, Sophie Vallet, Wesley H. Brooks, Yves Renaudineau

**Affiliations:** 1 INSERM ESPRI, ERI29/EA2216, SFR ScInBioS, LabEx IGO “Immunotherapy Graft Oncology”, Réseau épigénétique et réseau canaux ioniques du Cancéropole Grand Ouest, European University of Brittany, Brest 29609, France; E-Mail: yves.renaudineau@univ-brest.fr; 2 LUBEM (EA3882), University of Brest; Laboratory of Virology, CHRU Cavale Blanche, Brest 29200, France; E-Mail: sophie.vallet@chu-brest.fr; 3 Department of Chemistry, University of South Florida, Tampa, FL 33620, USA; E-Mail: wesleybrooks@usf.edu; 4 Laboratory of Immunology and Immunotherapy, CHRU Morvan, Brest 29609, France

**Keywords:** HERV-E, DNA methylation, cancer, autoimmunity

## Abstract

Human endogenous retrovirus group E (HERV-E) elements are stably integrated into the human genome, transmitted vertically in a Mendelian manner, and are endowed with transcriptional activity as alternative promoters or enhancers. Such effects are under the control of the proviral long terminal repeats (LTR) that are organized into three HERV-E phylogenetic subgroups, namely LTR2, LTR2B, and LTR2C. Moreover, HERV-E expression is tissue-specific, and silenced by epigenetic constraints that may be disrupted in cancer, autoimmunity, and human placentation. Interest in HERV-E with regard to these conditions has been stimulated further by concerns regarding the capacity of HERV-E elements to modify the expression of neighboring genes and/or to produce retroviral proteins, including immunosuppressive *env* peptides, which in turn may induce (auto)-antibody (Ab) production. Finally, better understanding of HERV-E elements may have clinical applications for prevention, diagnosis, prognosis, and therapy.

## 1. Introduction

Human endogenous retroviruses (HERV) are descendants of occasional germline invasion by exogenous retroviruses. HERVs occupy as much as 8% of the ~3 billion base pairs (bp) of the human genome [1,2]. Such elements, distributed in ~400,000 loci and transmitted vertically in a Mendelian manner, are categorized into 30–40 families depending on the method of classification [3,4]. Each family encompasses tens to thousands of loci [5], and several criteria are used for HERV classification, including (i) the single letter amino acid tRNA primer binding site used by the provirus; (ii) the similarity with animal retroviruses, which divides them into three classes (class I: gammaretrovirus; class II: betaretrovirus; and class III: spuma(retro)virus); and (iii) the molecular genetic means. Needless to say, a systematic nomenclature is necessary for naming all the retroviral elements present in the genome, but such a consensus nomenclature has not yet been established [6,7].

In their original form, HERVs possess two unique (U)3-repeat(R)-U5 long terminal repeats (LTR) present at the extremities, and flanking the three HERV genes: the group-specific antigen (*gag*), reverse transcriptase/polymerase (*pol*) and envelope (*env*) genes. The HERV *gag*, *pol* and *env* genes, when present, have the particularity of being disrupted by frame shift mutations, stop codons and/or deletions that affect their retroviral replicative capacity as well as their ability to transpose and to create *de novo* insertional mutations. Similarly, the regulatory sequences present in the LTRs have accumulated mutations, and most of them are transcriptionally silenced by epigenetic constraints [8].

This review will focus on the HERV group E family (HERV-E), which uses tRNA^Glu^ and which is related to class I gammaretrovirus based on homology with the Moloney Murine Leukaemia Virus (MoMuLV) *pol*, *gag* and *env* genes [9]. Interest in HERV-E elements has been stimulated further by concerns regarding their occurrence and implication in malignancies, autoimmune diseases, and human placentation.

## 2. The HERV-E Family

### 2.1. HERV-E Phylogeny

**Figure 1:**
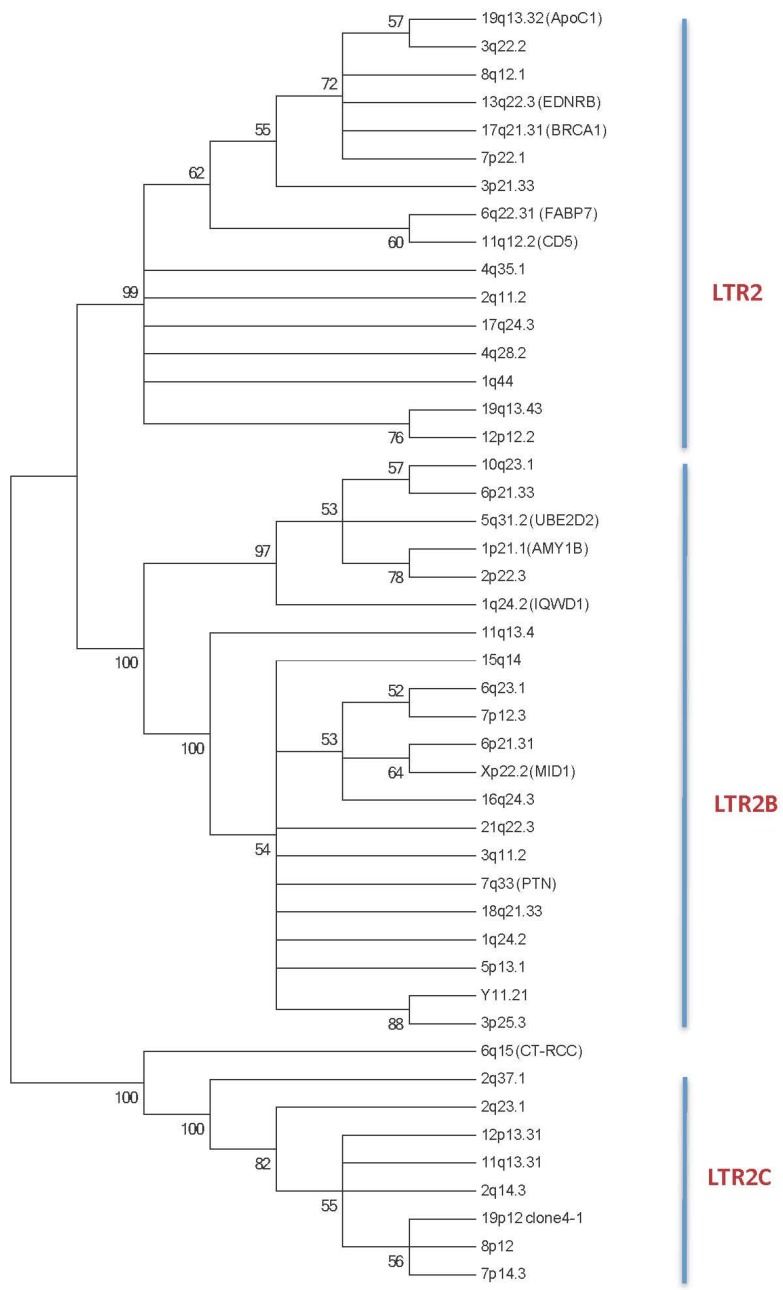
The phylogenetic tree of the 5’ LTR HERV-E sequences reveals three main subgroups: LTR2, LTR2B and LTR2C. Phylogenetic analyses were inferred using the Maximum Parsimony Methods on the ClustalW Multiple alignment of 46 HERV-E 5’LTR nucleotide sequences. The most parsimonious tree is shown; it shows that the 46 HERV-E LTRs are segregated into 3 distinct subgroups. Bootstrap values greater than 50% are shown. The 5’ HERV-E LTR sequences are identified with their respective human chromosomal locus location (See Experimental Section plus Table 1). When known, the gene used for alternative transcript usage is reported.

More than 1,300 HERV-E elements have been described in the human genome (DFAM 1.3 release December 2014, [7,10], and these elements are subdivided into three distinct subgroups referred to as LTR2 (*n* = 811, 60%), LTR2B (*n* = 302, 23%) and LTR2C (*n* = 223, 17%) based on divergences observed between sequences of the 5’LTR part of the HERV-E [11]. Similarities between the three subgroups range from 75% (LTR2B/LTR2C), to 81% (LTR2/LTR2C), and 83% (LTR2/LTR2B). To go further in the exploration of these three subgroups, we have updated our initial 5’LTR HERV-E phylogenic tree [12] with new sequences in order to build a new phylogenic tree containing 16/46 (34.8%) LTR2 sequences, 21/46 (45.6%) LTR2B sequences, and 9/46 (19.6%) LTR2C sequences (Table 1 and Figure 1). 

**Table 1:** Description of the 46 HERV-E elements explored (see Experimental Section).

Locus	HERV Name	Subgroup	5’LTR Genomic Location	3’LTR Genomic Location	Size (bp)	Structure	5’ and 3’LTR Divergences	Molecular Clock Dating	EPO Alignment Dating	Genomic Amplification
19q13.32	HERV-E.ApoC1	LTR2	19:44913964-44914947	-	987	solo LTR	-	-	>15 My	>15 My [ 65]
3q22.2	-	LTR2	3:134027135-134027667	3:134019829-134020359	7839	gag, pol truncated	9.5%	32 My	>15 My	
8q12.1	-	LTR2	8:58690826-58691341	8:58698386-58698895	8069	gag, pol truncated	7.0%	23 My	>15 My	
13q22.3	HERV-E.EDNRB	LTR2	13:77975613-77976074	13:77975589-77976108	5777	gag, pol, env truncated	49.7%	>50 My	>25 My	>25 My [ 65]
17q21.31	HERV-E.BRCA1	LTR2	17:43160221-43160752	17:43167223-43167735	7514	gag, pol truncated	9.4%	31 My	>15 My	
7p22.1	-	LTR2	7:100919594-100920108	-	514	solo LTR	-	-	>15 My	
3p21.33		LTR2	3:43678285-34678772	-	487	solo LTR	-	-	>15 My	
6q22.31	HERV-E.FABP7	LTR2	6:122748781-122749289	6:122741492-122741993	7752	pol truncated	9.0%	30 My	>25 My	
11q12.2	HERV-E.CD5	LTR2	11:61093564-61094073	11:61098368-61098863	5254	pol, env truncated	7.8%	26 My	>25 My	> 25 My [ 11]
4q35.1	-	LTR2	4:183377788-183378289	-	501	solo LTR	-	-	>25 My	
2q11.2	-	LTR2	2:101186575-101186124	-	451	solo LTR	-	-	-	
17q24.3	-	LTR2	17:71020856-71021358	-	502	solo LTR	-	-	>15 My	
4q28.2	-	LTR2	4:128703926-128704424	-	498	solo LTR	-	-	>25 My	
1q44	-	LTR2	1:247127397-247127939	-	542	Solo LTR	12.3%	41 My	>15 My	
19q13.43	-	LTR2	19:57815303-57815707	-	1163	solo LTR	-	-	>25 My	
12p12.2	-	LTR2	12:20944374-20944873	-	499	solo LTR	-	-	>15 My	
10q23.1	-	LTR2B	10:84172465-84171987	-	997	solo LTR	-	-	>15 My	
6q21.33		LTR2B	6:31186291-31186769	-	478	solo LTR	-	-	>8 My	
5q31.2	HERV-E.UBE2D2	LTR2B	5:139525866-139526346	5:139531087-139531583	5644	gag, pol truncated	5.2%	17 My	>15 My	
1p21	HERV-E-AMY1B	LTR2B	1:103696448-103696893	1:103703817-103704285	7837	full length	11.9%	40 My	>6 My	
2p22.3	-	LTR2B	2:34677243-34677662	-	888	solo LTR	-	-	>15 My	
1q24.2	HERV-E.IQWD1 (DCAF6, PC326)	LTR2B	1:167863096-167863572	1:167869427-167869928	6832	gag, pol truncated	6.9%	23 My	>15 My	
11q13.4	-	LTR2B	11:73271302-73271710	-	408	solo LTR	-	-	>15 My	
15q14	-	LTR2B	15:34812862-34813351	-	489	solo LTR	-	-	>15 My	
6q23.1	-	LTR2B	6:130260127-130260610	6:130267508-130267970	7843	gag, pol truncated	9.7%	32 My	>15 My	
7p12.3	-	LTR2B	7:46031148-46031644	-	496	solo LTR	-	-	>15 My	
6p21.31	-	LTR2B	6:35004307-35003824	-	483	solo LTR	-	-	>15 My	
Xp22.22	HERV-E.MID1	LTR2B	X:10585282-10584793	X:10590339-10589853	5,546	gag, pol, env truncated	6.4%	20 My	>15 My	> 25 My [ 55]
16q24.3	-	LTR2B	16:90056524-90057012	-	488	solo LTR	-	-	>15 My	
21q22.3	-	LTR2B	21:42807398-42807919	-	521	solo LTR	-	-	>15 My	
3q11.2	-	LTR2B	3:97856881-97857316	-	435	solo LTR	-	-	-	
7q33	HERV-E.PTN	LTR2B	7:136947291-136947711	7:137262486-137263044	6,360	gag, pol, env truncated	7.9%	26 My	>15 My	>15 My [ 11,20,40]
18q21.33	-	LTR2B	18:61617742-61618212	-	470	solo LTR	-	-	-	
1q24.2		LTR2B	1:167869414-167869892	-	478	solo LTR	-	-	>15 My	
5p13.1	-	LTR2B	5:40872315-40872812	-	497	solo LTR	-	-	> 15 My	
Yq11.21	-	LTR2B	Y:12245468-12245973	Y:12251519-12252038	6571	gag truncated	14.4%	48 My	-	
3p25.3	-	LTR2B	3:9590155-9590642	-	487	solo LTR	-	-	> 25 My	
6q15	CT-RCC HERV-E	LTR2C	6:88662137-88662613	6:88670478-88670972	8792	full length	5.8%	19 My	> 15 My	
2q37.1	-	LTR2C	2:231400694-231401230	2:231408328-231408867	8173	full length	2.9%	10 My	> 15 My	
2q23.1	-	LTR2C	2:149035320-149035863	-	543	solo LTR	-	-	> 8 My	
12p13.31	-	LTR2C	12:7703612-7704158	-	546	solo LTR	-	-	> 8 My	
11q13.31	-	LTR2C	11:66000626-66001172	-	546	solo LTR	-	-	> 8 My	
2q14.3	-	LTR2C	2:127683400-127683948	-	548	solo LTR	-	-	> 8 My	
19p12	HERV-E clone 4-1	LTR2C	19:20755113-20755654	19:20746795-20747340	8,806	full length	4.5%	15 My	> 8 My	
8p12	-	LTR2C	8:30728558-30729101	-	5,121	env, 3’LTR truncated	-	-	> 8 My	
7p14.3	-	LTR2C	7:32762447-32762988	-	541	solo LTR	-	-	> 6 My	

EPO: Enredo-Pecan-Ortheus whole genome primate alignment tool from ensembl.

#### 2.1.1. HERV-E Subgroups and Integration Time

Using both the phylogenic approach by genomic amplification with different primates, from New World monkeys to humans, and the molecular clock approach, Li *et al.* have established two waves in HERV-E lineage integration [13]. The molecular clock approach is used to date the age of a HERV-E element insertion by applying an evolutionary mutation rate of ~0.3% mutation per million years (My) [13,14]. The first insertion occurred after the divergence of the New World primates from the Old World primates ~45 My ago, and a second insertion started ~25 My ago with the beginning of hominoid evolution.

**Figure 2:**
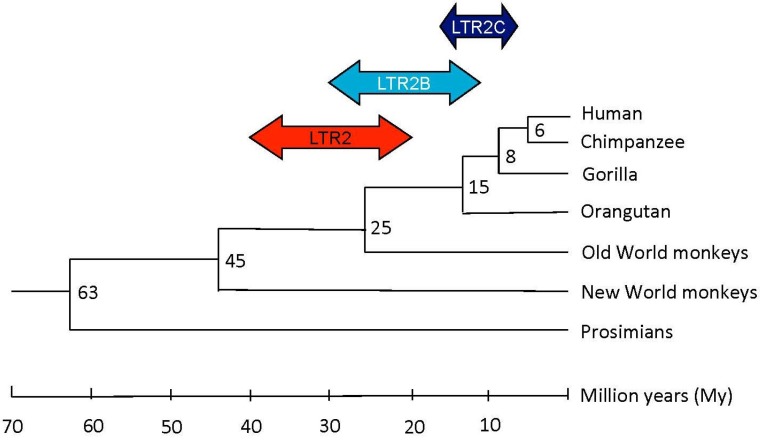
Putative integration times of the LTR2, LTR2B, LTR2C HERV-E familly members during primate evolution. The evolutionary tree was adapted from Yi *et al*. [12,13].

Applied to our selection of HERV-E elements, we can complete Li’s observations and establish that HERV-E integration has occurred in several waves according to the LTR2 subgroup (for more information, see the Experimental Section). As summarized in Figure 2 and reported in Table 1, integration for LTR2 occurred predominantly before the divergence of hominoids from Old World monkeys, for LTR2B at the time of hominoid divergence, and in the case of LTR2C elements throughout hominoid evolution.

#### 2.1.2. HERV-E Subgroups and Structure

We and others have established that HERV-E elements are disseminated within the genome as solitary LTR elements, HERV-E *gag*, *pol*, *env* and/or 3’LTR deleted provirus, while full length and mutated HERV-E elements were detected in a few cases (e.g., HERV-E clone 4-1) [12,15,16]. With regards to our selection of HERV-E elements (Table 1), solitary LTRs (*n* = 29/46, 63.0%) represent the main group, followed by truncated provirus (*n* = 13/46, 28.3%), and full length HERV-Es (*n* = 4/46, 8.7%).

#### 2.1.3. HERV-E Subgroups and Promoter Activity

Basically, the consensus sequences of LTR2 (463 bp, DF0000449), LTR2B (483 bp, DF0000484) and LTR2C (501 bp, DF0000485) [10] are composed of three parts: a transcriptional control U3 region, a transcription initiation site which defines the U3/R boundary at positions 298 to 337, and a poly-adenylation signal which defines the R/U5 boundary at positions 399 to 435. 

The alignment analysis of the U3, R and U5 parts reveals important variations between LTR2, LTR2B and LTR2C with similarities ranging from 57.0% to 71.8% in U3; 73.5% to 87.8% in R; and 84.4% to 93.7% in U5. As a consequence, the differences observed in the regulatory U3 region between the three subgroups suggest distinct transcription binding sites and promoter activity between LTR2, LTR2B and LTR2C elements. This assertion is reinforced by the observation that placentally expressed LTR2B (HERV-E.PTN, HERV-E.MID1, and HERV-E PC326/IQWD1) shared >88.0% homology in U3, and that B-cell expressing LTR2 provirus (HERV-E.CD5 and HERV-E.FABP7) shared 94.0% homology in U3. Further analyses are required to characterize consensus transcription factor binding sites in each subgroup.

#### 2.1.4. Chromosomal Integration and Consequences

Analysis of the DFAM 1.3 database for the presence of HERV-E in the human genome reveals HERV-E sequence integrations on all chromosomes with a preferential distal position. Furthermore, recent technological advances in sequencing and bioinformatics have further established a predominant HERV intergenic integration and an antisense orientation to the host gene containing the HERV with a ratio of 3:1 for HERV-E elements [17]. Such observations suggest that HERVs, when integrated in the same orientation and/or present in the introns, are more likely to be deleted to prevent a negative effect on the neighbouring or integrated gene.

**Figure 3:**
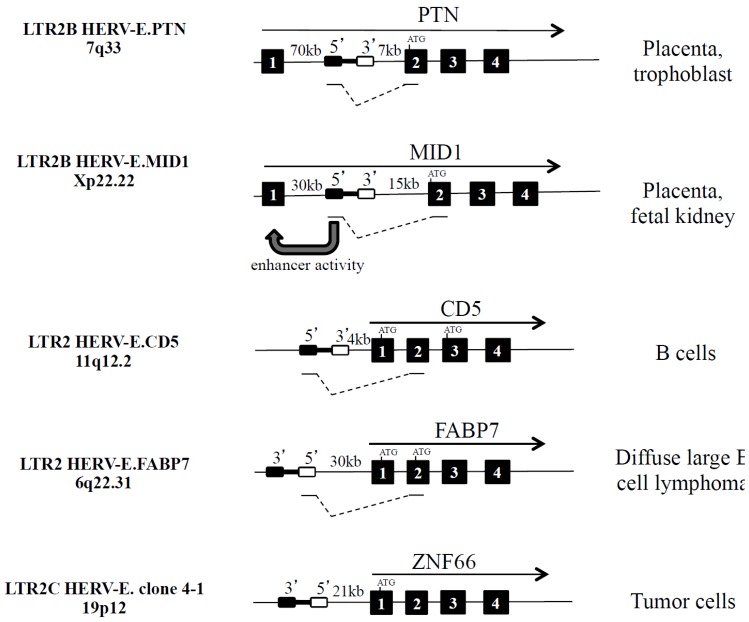
Examples of five integrated HERV-Es in the vicinity of PTN, MID1, CD5, FABP7 and ZNF66 human genes. HERV-E is depicted as a black rectangle (5’LTR) linked to a white rectangle (3’LTR). The direction of gene transcription is indicated (black arrow). Black boxes refer to exons, and fusion transcripts are indicated.

Analysis of the human Expressed Sequence Tag (EST) database reveals an important proportion of HERVs and LTR elements in the transcriptome and, among them, HERV-Es are overrepresented and initiate 9.3% of the chimeric LTR transcripts when leukemic B cells from diffuse large B cell lymphoma are analyzed [18]. In general, when the HERV element is in the sense strand, upstream of the host promoter (exon 1) or in the first intron, the HERV element can act as an alternative promoter, splice with exon 2, and generate a fusion mRNA with a longer 5’ untranslated region (UTR). The splicing of the HERV element to a splice acceptor site in the host gene sequence could shift the open reading frame (ORF), yielding a transcript that cannot be translated into the functional host gene product as observed with HERV-E.CD5 (Figure 3). In contrast, when HERV elements are in the anti-sense strand relative to neighbouring host genes, the active HERV element could either disrupt expression of the host genes, dampening the host gene activity or act as a promoter (e.g., HERV-E.FABP7) or an enhancer (e.g., HERV-E.AMY1B [19,20]) to promote an alternative tissue expression. When the HERV element is in the body of a host gene, the HERV can provide additional splice donor site(s), acceptor site(s), or premature polyadenylation signals resulting in creation of incorrectly spliced and/or truncated variants.

Last but not least, HERV-E transcripts can also arise from the HERV-E element, and transcripts containing *gag*, *pol* and/or *env* genes may be expressed as observed with the HERV-E clone 4-1 [16]. The translated products from HERV-E genes could have antigenic potential (*gag* and *env* peptides), and/or immunosuppressive functions (*env* peptides).

### 2.2. Examples

#### 2.2.1. Alternative Promoter: LTR2B HERV-E.PTN and LTR2B HERV-E.MID1

As indicated in Figure 3 and Table 2, both HERV-E.PTN and HERV-E.MID1 are integrated in the same orientation and upstream from the second exon of the secreted heparin-binding cytokine pleiotrophin (PTN) on one hand [21,22] and the microtubule associated protein Mid1 that targets the phosphatase 2A protein for degradation on the other hand [23]. Mid1 is involved in the pathogenesis of the X-linked form of Opitz syndrome when mutated [24]. For these two HERV-E elements, the presence of HERV-E pseudogene deletions (*gag*, *pol* and *env)* and an insertion of a truncated RTLV-Ib element (*pol* and *env*) were reported [21,22].

For the LTR-PTN and the LTR-Mid1 transcripts, the ORF, present in exon 2, is conserved, and as a consequence, differences are related to the acquisition of a cell-specific expression in the placenta. Furthermore, it was also observed that the 5’ LTR2B part of HERV-E.MID1 participates in the regulation of the non-retroviral Mid1 promoter as an enhancer in placental cells [24].

**Table 2:** HERV-E integration influences human genes.

Locus	Gene Name (Function)	Subgroup	Gene Location	Orientation	Transcripts	Chimeric Transcript	Protein Variant	Reference
1p21	AMY1B (salivary amylase)	LTR2	Upstream	antisense	salivary	No (enhancer)	No	[18,19]
1q24.2	IQWD1/DCAF6 (nuclear receptor)	LTR2B	Upstream	sense	placenta	Yes	unknown	[11,40]
7q33	PTN (secreted heparin-binding cytokine)	LTR2B	Intron 1	sense	Placenta, trophoblast	Yes	No	[11,20,40]
5q31.2	UBE2D2 (ubiquitin conjugating enzyme)	LTR2B	Upstream	sense	unknown	Yes	No	[11]
11q12.2	CD5 (T cell and B cell subset antigen)	LTR2	Upstream	sense	B cells	Yes	Yes	[11,66]
13q22.3	EDNRB (G protein-coupled receptor of endothelin)	LTR2	Upstream	sense	placenta	Yes	No	[11,40,65,67]
19q13.32	ApoC1 (lipoprotein metabolism)	LTR2	Upstream		Liver, macrophage	Two variants	No	[65]
6q22.31	FABP7 (brain lipid binding protein)	LTR2	Upstream	antisense	B cell lymphoma	Yes	Yes	[17]
Xp22.22	MID1 (microtubule-associated protein)	LTR2B	Intron 1	sense	Placenta, fetal kidney	Yes	No	[11,23,40,67]

#### 2.2.2. Alternative Variants: HERV-E.CD5 and HERV-E.FABP7

The 5254 bp retroviral sequence HERV-E.CD5 is integrated into chromosome 11 at position 11q12.2 within the *cd5* gene locus and 5 kilobases (kb) upstream of the host CD5 gene exon 1, in the same transcriptional direction, and downstream of the *cd6* gene [25]. Within the two LTRs, the *gag* gene is present while the *pol* gene and the *env* gene, in part, are lacking. Using the molecular clock approach (5’ and 3’ LTR2 identity is 92.2%), and a panel of hominoids, Old World monkeys, and New World monkeys, we have established that HERV-E.CD5 integration occurred just prior to the divergence of hominoids from Old World monkeys ~25 My ago [12].

In human B cells, HERV-E insertion introduces another promoter for the *cd5* gene, and enables transcription of a fusion transcript that splices in CD5 exon 2 with the consequence of an ORF shift to exon 3, leading to an intracellular variant of CD5 [26]. The LTR2-CD5 fusion transcripts are found in lymphoid organs, and such expression is confined to B cells with the higher expression observed in the B1 cell subset [27,28]. We have not detected proviral HERV-E.CD5 transcripts containing *gag* and/or *env* genes.

As recently described, the HERV-E.FABP7 element was observed to drive a new isoform in diffuse large B cell lymphoma [18]. Inserted 30 kb upstream of the *fabp7* gene locus in the antisense orientation, the HERV-E.FABP7 element creates an alternative promoter which produces an ectopic LTR2-driven chimeric transcript. The LTR2-FABP7 transcript skips the normal ORF site from exon 1 to exon 2 (Figure 3). The contribution from the three conserved residues of the nuclear localisation signal (NLS) present in exon 1 is lost. As a consequence, the chimeric FABP7 protein cannot translocate to the nucleus and cannot exert its repressive action on leukemic B cell proliferation.

#### 2.2.3. Proviral Proteins: LTR2C HERV-E Clone 4-1

Originally isolated by Martin *et al.* [29] and sequenced by Repaske *et al.* [30], the 8806 (bp) HERV-E clone 4-1 is inserted in the short arm of chromosome 19 at position 19p12 upstream of the *znf66* gene locus and in the antisense orientation. This full-length HERV-E is considered to be an LTR2C prototype containing 5’ and 3’ LTR elements that are 95.5% identical and encompass *gag*, *pol* and *env* genes. HERV-E clone 4-1 contains nucleotide substitutions and deletions that introduce stop codons and changes in the ORF, thereby precluding its capacity to form infectious viral particles. However, HERV-E clone 4-1 has long ORFs in the *gag* and e*nv* regions that can effectively encode a p30gag protein and a p15env protein [31]. The HERV-E clone 4-1 exhibits approximately 40% homology with the murine ancestor MoMuLV.

### 2.3. DNA Methylation Controls Transcription

HERV elements are tightly controlled by DNA methylation [32,33,34]. As a consequence, several authors have used HERV-E elements as a sensor of the DNA methylation state in response to external stimuli and/or in pathological situations to test for possible epigenetic deregulation. Epigenetics is defined as mechanisms by which stable and heritable changes in DNA methylation and packaging of genes can control transcription without affecting the underlying DNA sequences (reviewed in [35,36]). The epigenetic machinery includes the control of DNA methylation, which involves the addition of a methyl group at position 5 of the cytosine pyrimidine ring to form 5-methylcytosine (5mC) within CpG sites. The DNA methylation reaction is controlled by DNA methyltransferases (DNMTs) which use S-adenosylmethionine (SAM) as the methyl donor.

Analysis of the CpG sites in the 5’ LTR2 of HERV-E.CD5 using methylation-sensitive endonuclease assays followed by polymerase chain reaction and bisulfite sequencing revealed that U3 CpG motifs are hypomethylated in B cells expressing LTR2-CD5 fusion transcripts [37]. In addition to these observations, our studies have revealed that cytokines, such as IL-6, may be involved in influencing the HERV-E.CD5 U3 DNA methylation status, and, in turn, LTR2-CD5 expression. Interestingly, LTR2-CD5 overexpression is effective in inducing cytokine expression, such as IL-5, IL-10 and IL-13, and in controlling the cell surface expression of the host gene which controls the negative selection of autoreactive B cells, raising the possibility that HERV-E.CD5 participates in an autoimmune response process [38,39,40].

Cell-specific LTR-promoter DNA demethylation leading to expression of the HERV-E transcripts is not restricted to HERV-E.CD5. It has also been described for HERV-E.FABP7 in leukemic B cells [18]; HERV-E.PTN, HERV-E.EDNRB, and HERV-E.MID1 in placenta but not in blood cells [41]; and HERV-E clone 4-1 in peripheral blood lymphocytes (CD4^+^, CD8^+^ and B cells) but not in neutrophils from patients with systemic lupus erythematosus (SLE) [42,43,44]. SLE is regarded as the prototype of autoimmune diseases.

## 3. HERV-E and Diseases

HERV-Es have attracted significant attention due to their association with malignancies, autoimmune diseases, and human placentation. Interest has been stimulated further by concerns regarding (i) their capacity to produce retroviral protein and in turn to induce (auto)-antibody (Ab) production; (ii) the immunosuppressive functions of the retroviral *env* proteins; (iii) their capacity to act as alternative promoters or enhancers; (iv) and their DNA methylation dependence.

### 3.1. Cancer

HERV-E expression has been reported in several tumour cells, such as those in human mammary glands and the prostate, ovary, colon, germinal cells, and uterus [45,46]. Regarding the HERV-E clone 4-1, its *env* transcript was reported in prostate, ovary and uterus cancer but not in healthy controls [31]. As a consequence, Abs directed against the p15env clone 4-1 peptide were further detected by ELISA in 40% of the sera obtained from women with ovarian cancer but not from normal controls [31]. Anti-p15env Abs are effective in inhibiting the immunosuppressive activity of the p15env clone 4-1 peptide [47]. The immunosuppressive effect of retroviral *env* peptides is also observed with other HERVs (e.g., HERV-K/HML-2 and HERV-R/ERV3), and that effect is related to the presence of a conserved immunosuppressive retroviral domain located within the plasma-membrane insertion part of the *env* protein [48].

In renal cell carcinoma (RCC), but not in normal tissues, the LTR2C full length CT-RCC HERV-E element located on chromosome 6 at position 6q15 has been shown to encode a highly immunogenic 10 amino acid *env* peptide (CT-RCC-1) that can be considered to be a tumour-specific antigen [49]. The CT-RCC-1 *env* peptide was found to promote RCC reactive CD8^+^ T cells and RCC cytotoxic T cells as observed in one patient with RCC. Expression of this proviral *env* peptide is controlled by DNA methylation at LTR sequences, and transcription is under the positive control of the HIF-2α transcription factor, which is itself repressed by the von Hippel-Lindau tumor-suppressor gene [50].

### 3.2. Autoimmune Diseases

Enhanced expression of mRNA from HERV-E clone 4-1 (M10976) was reported in T cells from patients with SLE [51], and in salivary glands isolated from patients with Sjögren’s syndrome [52,53]. A repressive effect of UVB and steroid treatment on the expression of HERV-E clone 4-1 was also observed in cultured human keratinocytes and in CD4^+^ T cells from SLE patients, respectively [54,55]. Interestingly, a positive correlation was reported between the 5’ LTR2C clone 4-1 demethylation status in CD4^+^ T cells with SLE activity, leukopenia and lymphopenia [42].

Abs directed against clone 4-1 p30gag protein were detected in 48% of SLE patients, 35.0% of patients with Sjögren’s syndrome, 33.3% of patients with mixed connective disease, and none of the healthy controls tested [56]. Anti-p30gag Abs also cross-react with two nuclear autoantigens used in the diagnosis of autoimmune diseases, U1-RNP and Sm autoantigens [57]. As a consequence, anti-p30gag Abs are believed to participate in the formation of immune complexes that, in turn, contribute to the activation in the complement pathway in the target organs. The effects of the clone 4-1 p15env protein were further tested *in vitro,* revealing, on one hand, its capacity to induce T cell activation and anergy, and, on the other hand, its capacity to induce IL-6 and IL-16 cytokine production [58].

Another SLE susceptible endogenous gammaretrovirus is the non-HERV-E human T cell leukaemia-related endogenous retrovirus (HRES-1) that is inserted in the long arm of chromosome 1 at position 1q42 [59]. HRES-1 possesses several similarities with HERV-E clone 4-1: (i) epigenetic control by DNA methylation [60]; (ii) the capacity to produce a p38gag protein that can, in turn, induce the development of Abs as observed in 52% of patients with SLE and in contrast to 3.6% in healthy donors [61]; and (iii) a cross-reactivity of the anti-p38gag HRES-1 Ab with the nuclear autoantigen U1-RNP.

### 3.3. Human Placentation

In placenta, several studies have highlighted the contribution of various HERV proviruses in normal placenta development and in maintenance of foetomaternal tolerance. The placenta, and particularly the syncytiotrophoblasts, shows expression of several HERVs, such as the ERVW-1 and ERVFRD-1 loci (syncytin-1 and syncytin-2) which are located on chromosomes 7 and 6, respectively [62], and HERV-E proviruses that function as tissue-specific and alternative gene promoters for PTN (growth function), Mid1 (placental development), ApoC1 (lipid metabolism) and EDNRB (anti-apoptotic activity) and appear essential to placental development and function [21,22,23]. HERV-E provirus expression results from a lower DNA methylation state in the placenta, ranging from 4% to 91% when analysing the HERV-E LTRs [41]. Other factors, such as transcription factors, are also involved for cellular specificity. This assertion is reinforced by the characterization of an Sp1 binding site present in the 5’ LTR of HERV-E.PTN, HERV-E.ApoC1 and HERV-E.EDNRB that was found to be critical for strong HERV-E placental transcriptional activity [22,63].

## 4. Experimental Section

Starting from the HERV-E elements characterized in our previous study [12], we have (i) performed a new selection of HERV-E elements using the online basic local alignment search tool (human BLASTN) provided by ensembl [64] ; (ii) retrieved the 5’LTR and 3’LTR proviral chromosomal locations and HERV-E sizes for the 46 selected HERV-Es using the DFAM 1.3 database [10]; (iii) performed an analysis of HERV-Es relative to sequences from HERV-E clone 4-1 in order to characterize *gag*, *pol* and/or *env* deletions using the dot plot matrix tool [65]; (iv) performed an alignment analysis to test the divergences among U3, R and U5 parts between LTR2, LTR2B and LTR2C subgroups [66]; and (v) used the ensembl genome browser for positioning the HERV-E elements in the human genome. When known, the gene used for alternative transcript usage was used to name the HERV-E element.

Phylogenetic reconstructions were conducted in MEGA 6 [67] with the Maximum Parsimony (MP) approach with a bootstrap resampling of 1000; they were inferred on 46 HERV-E 5’LTR nucleotide sequences aligned using the ClustalW Multiple Alignment method of BioEdit version 7.2.5. There were a total of 637 positions in the final dataset. The MP tree was obtained using the Subtree-Pruning-Regrafting (SPR) algorithm [68] with search level 1, in which the initial trees were obtained by the random addition of sequences.

To date HERV-E integration, three approaches were selected: (1) molecular clock using divergences between 5’LTR and 3’LTR [66] and an evolutionary rate of 0.3% mutation per My [13,14]; (2) whole-genome alignments using the eight-primate Enredo-Pecan-Ortheus (EPO) alignment tool generated by ensembl to determine the integration time during primate evolution (>6 My: Chimpanzee, >8 My: Gorilla, >15 My: Orangutan, >25 My: Old World monkeys, i.e. vervet-AGM, macaque and olive baboon; and >45 My: Marmoset as New World monkeys); and (3) genomic amplification when reported in the literature.

## 5. Conclusions

The widespread distribution of HERV elements near human gene promoters in the human genome implies a large panel of putative biological activities. The results that may occur can include truncation of host gene products; improper localization of host gene products due to missing localization signals; and ectopic increased or decreased expression of the host gene. The HERV elements can also, from their own sequence, generate antigenic material, and produce immunosuppressive *env* peptides. Almost all these effects have been reported for HERV-E elements in pathological conditions and/or during placental formation, supporting an important contribution for this family to particular disease states and human evolution. The HERV-E family is also characterized by its heterogeneity, which is in part related to the presence of three main subgroups.

To date, most of the studies testing HERV-E elements in human diseases consider the HERV-E family a minor group with few members, and the HERV-E clone 4-1 as the prototype for HERV-E. However, such assertions need to be revised based on the observations that the HERV-E family possesses at least 1300 members and that HERV-E clone 4-1 belongs to the LTR2C HERV-E subgroup, which is distinct from the two other subgroups in terms of regulation and integration time in the human genome. As a consequence, we recommend, for future studies, using a panel of HERV-Es selected from the three subgroups.

According to the growing importance of HERV-E elements in human diseases, the next challenges are related to acquiring more details about the characterization of HERV-E regulation, HERV-E insertion sites, HERV-E (fusion) transcripts, and HERV-E peptide production, specifically focusing on the antigenic *gag* peptides and the immunosuppressive *env* peptides. Altogether, a better understanding of the HERV-E elements and their impact on cellular biology will have applications in the development of new biomarkers for prevention, diagnosis, prognosis, and therapy.
